# Deposit-Feeding Sea Cucumbers Enhance Mineralization and Nutrient Cycling in Organically-Enriched Coastal Sediments

**DOI:** 10.1371/journal.pone.0050031

**Published:** 2012-11-27

**Authors:** Thomas MacTavish, Jeanie Stenton-Dozey, Kay Vopel, Candida Savage

**Affiliations:** 1 Department of Marine Science, University of Otago, Dunedin, New Zealand; 2 National Institute of Water and Atmospheric Research (NIWA), Riccarton, Christchurch, New Zealand; 3 School of Applied Sciences, Auckland University of Technology, Auckland, New Zealand; University of Southampton, United Kingdom

## Abstract

**Background:**

Bioturbators affect multiple biogeochemical interactions and have been suggested as suitable candidates to mitigate organic matter loading in marine sediments. However, predicting the effects of bioturbators at an ecosystem level can be difficult due to their complex positive and negative interactions with the microbial community.

**Methodology/Principal Findings:**

We quantified the effects of deposit-feeding sea cucumbers on benthic algal biomass (microphytobenthos, MPB), bacterial abundance, and the sediment–seawater exchange of dissolved oxygen and nutrients. The sea cucumbers increased the efflux of inorganic nitrogen (ammonium, NH_4_^+^) from organically enriched sediments, which stimulated algal productivity. Grazing by the sea cucumbers on MPB (evidenced by pheopigments), however, caused a net negative effect on primary producer biomass and total oxygen production. Further, there was an increased abundance of bacteria in sediment with sea cucumbers, suggesting facilitation. The sea cucumbers increased the ratio of oxygen consumption to production in surface sediment by shifting the microbial balance from producers to decomposers. This shift explains the increased efflux of inorganic nitrogen and concordant reduction in organic matter content in sediment with bioturbators.

**Conclusions/Significance:**

Our study demonstrates the functional role and potential of sea cucumbers to ameliorate some of the adverse effects of organic matter enrichment in coastal ecosystems.

## Introduction

Bioturbators enhance ecosystem functioning in marine sediments by modifying geochemical gradients, redistributing food resources [1], and altering nutrient fluxes. All of these factors influence coastal productivity [2]. Accordingly, the net effect of bioturbators on sediment biogeochemical processes is often markedly different from that predicted by simple linear food-chain theory [1]. Yet predicting ecosystem-level effects of these ‘ecosystem engineers’ can be challenging owing to multiple feedbacks and emergent properties [3], [4]. Accordingly, the potential consequences of losing these important functional groups from overfishing [5] or physical disturbance of the benthos [6] has ramifications for ocean ecosystems and human well-being. Global invertebrate catches have increased six-fold since 1950 [5], with overfishing causing population declines in 81% of sea cucumber fisheries [5], a ubiquitous and functionally important group of bioturbators [1]. Given their important role as bioturbators, it has been suggested that the presence of deposit-feeding sea cucumbers can reduce the accumulation of excess organic matter in coastal sediments such as under aquaculture farms [7], [8]. Furthermore, incorporating sea cucumbers into the aquaculture of finfish or bivalves may reduce pressure on overexploited wild populations and add another valuable farmed resource.

Marine aquaculture is expanding rapidly to meet global food demands, with the production of farmed fish reaching 50% of total fisheries production in 2009 [9]. In parallel with this increased production, however, comes a threat to marine ecosystems from excessive deposition of organic material to the seabed. The addition of organic matter, either as fish food and feces, or bivalve feces and pseudo-feces, alters benthic oxygen demand [10] and the structure and function of the benthos [11]. Benthic environments contribute both directly (primary and secondary production) and indirectly (through nutrient regeneration) to ecosystem energetics [12] and provide habitat for benthic organisms and many pelagic organisms at various stages of their lifecycle [13]. Thus, maintaining the resilience of benthic habitats around aquaculture farms is a key goal and challenge for resource managers [14].

Integrated multitrophic aquaculture (IMTA), whereby farm nutrients and organic particulates are recycled through the co-culture of species from different trophic levels, has been proposed to reduce production costs and minimize deleterious ecological effects of aquaculture [15]. However, in spite of research into fish–bivalve–seaweed combinations [16], the potential role of bioturbating benthic organisms in IMTA systems has received only limited attention. Deposit-feeding sea cucumbers reduce the accumulation of organic carbon under mussel farms [8] and thus show promise as a management tool; however the complexity of ecosystem functions performed by sea cucumbers demands a better understanding of their interactions with sediment biogeochemistry and benthic-pelagic coupling.

Most sea cucumbers are omnivorous deposit-feeders that not only compete with, but also feed directly upon, benthic bacteria [17]. Traditional food web models predict that bacteria, as the intermediate consumer, will eventually be excluded in such omnivorous associations [18]. However, the importance of positive interactions/facilitation [19] and scale niche differentiation [20] demonstrate that these traditional models have overlooked complex interactions among species. The stimulatory effects of sediment infauna on bacteria (e.g., increased O_2_ supply and release of reduced metabolites) are well understood [21] and may also be relevant to epibenthic organisms such as sea cucumbers. Moreover, by consuming and reconstituting large quantities of sediment, deposit-feeding sea cucumbers may create favorable conditions for bacterial activity [22]. Sediment feeding sea cucumbers also elevate nutrient concentrations through excretion [23], while bioturbation by burrowing *Echinocardium* can enhance nutrient efflux from the sediments [2]. The elevated nutrients in turn improve conditions for sediment primary producers, notably microphytobenthos (MPB) [2], [24]. However, deposit-feeding sea cucumbers also graze and through bioturbation can disturb MPB communities [25]. Moreover, positive and negative interactions can be dynamic, varying in their relative strength along environmental gradients [26]. The balance between organic matter (OM) remineralization by bacteria and nutrient assimilation by primary producers determines the rate of benthic-pelagic nutrient exchange and organic matter content in marine sediments [27]. Thus, the effect of bioturbation on microbial producers and decomposers has important consequences for the potential of sea cucumbers to ameliorate the effects of eutrophication.

In this study, we investigate the functional role of deposit-feeding sea cucumbers as bioturbators and quantify their effect on benthic algal biomass (chlorophyll-*a* of MPB), bacterial abundance, and the sediment–seawater exchange of dissolved oxygen and nutrients. We hypothesized firstly, that sea cucumbers would facilitate OM decomposition through enhanced bacterial abundance. Secondly, we hypothesized that producer (MPB) biomass would initially increase in the presence of bioturbators due to increased efflux of nutrients from the sediment, but that grazing by the sea cucumbers results in no net overall increase in algal biomass. We used the endemic deposit-feeding sea cucumber *Australostichopus mollis*, which is common on subtidal sediment throughout New Zealand and is of commercial interest as both a quota and bycatch fishery. Sea cucumbers were exposed to sediment enriched with feces and pseudofeces from the Greenshell™ mussel *Perna canaliculus*, the dominant aquaculture species in New Zealand.

## Results

Biomass of the sea cucumbers increased by 1.6% on average over the 14-day experiment. Sediment was predominantly mud (<0.63 µm) and contained relatively high densities of macrofauna (20–30 g w/w m^−2^), typical of that found in sheltered areas in the Marlborough Sounds [28], [29].

### Sediment Characteristics

**Figure 1:**
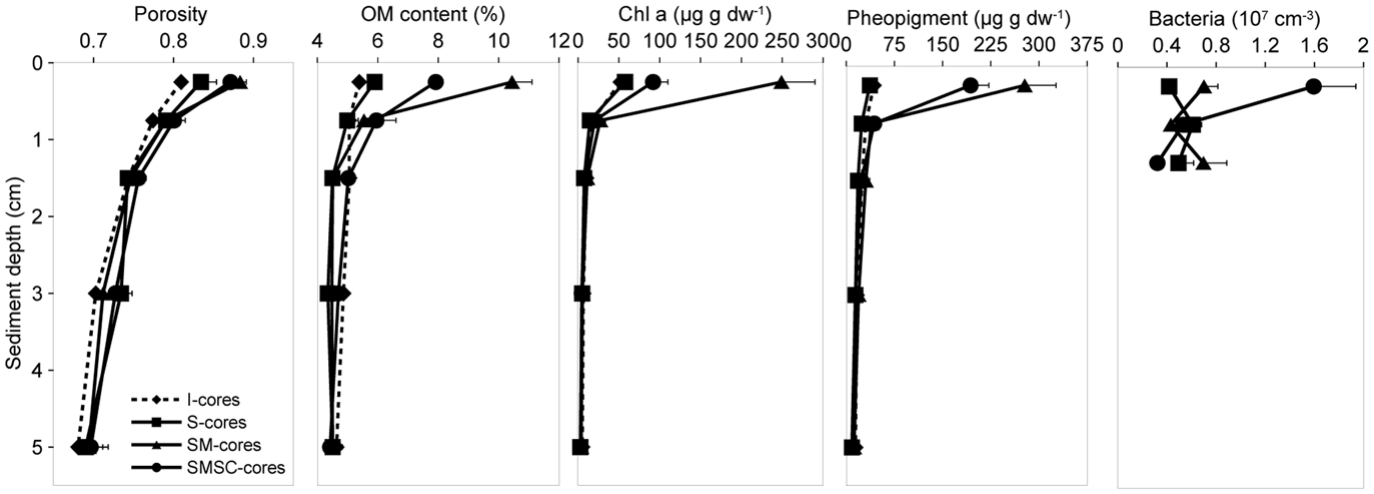
Mean OM content (%), chlorophyll a and pheopigment concentrations (µg **g** **dw** ^−**1**^ **) and bacterial abundance (10^7^** **cells** **cm** ^−**3**^ **) at intervals in the top 5 cm of sediment.** Error bars represent the standard error about the mean in the positive direction only.

Porosity of the top 0.5 cm of sediment was significantly higher in cores with added mussel biodeposits (SM-cores) and biodeposits plus sea cucumbers (SMSC-cores) compared to initial (I-cores) and control cores (S-cores) (Table 1). No other significant differences in porosity were detected at any interval. Organic matter (hereafter, OM) content was greatest in the cores with mussel feces (SM-cores), with 10.4±0.7% recorded in the top 0.5 cm after 14 days (Figure 1). This was significantly greater than OM content in the cores with mussel feces and sea cucumbers (SMSC-cores) for the same period (7.9±0.7%) (Table 1). The OM content in surface sediment of I and S-cores was 5.4±0.5% and 5.9±0.2% respectively, significantly lower than for SM- and SMSC-cores. Below 0.5 cm, the only significant differences were between I and S-cores at 1–2 and 2–4 cm.

**Table 1:** Summary of statistical analyses. ‘Repeated Measures ANOVA’ is abbreviated to ‘RM ANOVA’ in the table.

	Parameter/treatment	*F*	df (treatment, error)	*p*	Post hoc
General sediment characteristics	Porosity	23.7	3, 11	<0.001	SMSC = SM>I = S
(0–0.5 cm)	Organic matter	28.98	3, 12	<0.001	SM>SMSC>I = S
	Chorophyll a	19.05	3, 10	<0.001	SM>SMSC = I = S
	Pheopigment	64.94	3, 9	<0.001	SM = SMSC>I = S
	Total chloro-pigment	57.13	4, 16	<0.001	*A. mollis* feces = mussel feces = SM>SMSC
	Bacterial abundance	12.36	2, 9	0.003	SMSC>SM = S
Nutrient exchange pre test	NH_4_^+^ solute exchange	1.1	3, 11	0.389	
(One-way ANOVA)	NO_x_ solute exchange	2.36	3, 12	0.123	
	PO_4_^3−^ solute exchange	0.53	3, 12	0.673	
NH_4_^+^ exchange	experimental day	6.91	6, 88	<0.001	Day 2 differed from Days 4–14
(RM ANOVA)	light/dark	2.39	1, 88	0.126	
	treatment	21.54	2, 8	0.001	SMSC>SM = S
	experimental day*light/dark	10.61	6, 88	0.001	
	experimental day*treatment	3.16	12, 88	0.001	
NO_x_ exchange	experimental day	6.78	6, 116	<0.001	Day 14 differed from Days 2–12
(RM ANOVA)	light/dark	0.13	1, 116	0.715	
	treatment	3.43	2, 9	0.078	
	experimental day*light/dark	2.82	6, 116	0.014	
PO_4_^3−^ exchange	experimental day	1.22	6, 116	0.303	Day 14 differed from Days 2–12
(RM ANOVA)	light/dark	4.44	1, 116	0.037	
	treatment	2.58	2, 9	0.13	
	experimental day*treatment	3.46	12, 116	<0.001	
	experimental day*treatment*light/dark	4.5	12, 116	<0.001	
PO_4_^3−^ exchange (RM ANOVA)	experimental day	3.63	5, 98	0.005	Day 8 differed from Days 2 and 4
(excluding Day 14)	treatment	8.1	2, 9	0.01	SMSC = SM, SMSC>S, SM = S
	experimental day*light/dark	3.69	5, 98	0.004	
	experimental day*treatment	2.84	10, 98	0.004	
TOE (RM ANOVA)	light/dark	169.92	1, 6	<0.001	
TOE (One-way ANOVA)	treatment (dark hours)	27.82	2, 6	0.001	SM = SMSC>S
	treatment (light hours)	5.39	2, 6	0.046	SMSC = SM, SMSC>S, SM = S
OPD (RM ANOVA)	light/dark	56.55	1, 12	<0.001	
	treatment	31.16	3, 12	<0.001	SMSC = SM<I = S
*R* (RM ANOVA)	light/dark	61.16	1, 12	<0.001	
*R* (One-way ANOVA)	tr. (dark hours)	7.81	3, 12	0.004	S = I, SM, SM>I, SMSC = SM, SMSC>I, S
	tr. (light hours)	0.224	3, 12	0.878	

Chlorophyll *a* (chl *a*) and pheopigment concentrations were greatest in the sediment surface (top 0.5 cm) across all treatments (Figure 1). The mean chl *a* concentration in the surface of SM-cores was >200 µg g dw^−1^, significantly greater than concentrations in I, S, and SMSC-cores (mean: 50–100 µg g dw^−1^) (Table 1). I, S, and SMSC-cores did not differ significantly in chl *a* concentration. Pheopigment concentrations in the top 0.5 cm were significantly greater in SM-cores (278±49 µg g dw^−1^) and SMSC-cores (194±46 µg g dw^−1^) compared to I and S-cores (39±2 µg g dw^−1^ and 37±4 µg g dw^−1^ respectively). There were no significant differences among treatments in either chl *a* or pheopigment concentrations below 0.5 cm.

**Figure 2:**
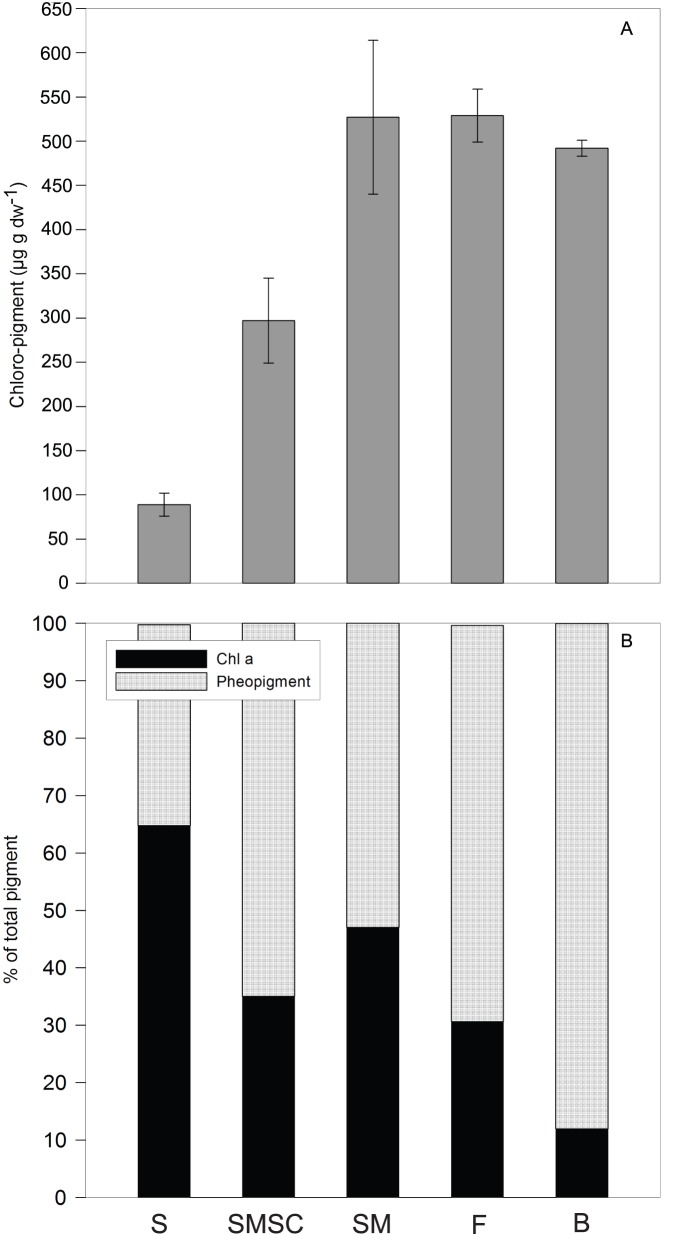
Pigment concentrations in the top 0.5 cm of sediment in S, SM and SMSC-cores, *A. mollis* feces (F) and mussel biodeposits (B). (A) The total concentration of chloro-pigment (µg g dw^−1^) and (B) the mean proportion (%) of chlorophyll *a* and pheopigment. Error bars represent the standard error about the mean.

Total chloro-pigment concentrations in *A. mollis* feces (529±30 µg g dw^−1^) were significantly greater than concentrations in the top 0.5 cm of SMSC-cores (297±48 µg g dw^−1^) (Table 1, Figure 2). There was, however, no significant difference in total chloro-pigment between *A. mollis* feces, mussel feces (492±19 µg g dw^−1^), and the top 0.5 cm of SM-cores (527±87 µg g dw^−1^). Although the mean chl a concentration of 162±23 µg g dw^−1^ in *A. mollis* feces was greater than in the top 0.5 cm of SMSC-cores (91±18 µg g dw^−1^), the ratio of chl a to total chloro-pigment was not significantly different (31% compared to 35%) (Figure 2). The proportion of chl *a* in chloro-pigment was 12% in mussel biodeposits, 48% in SM-cores, and 65% in S-cores. Given that chl a is less stable than pheopigment, the estimated 12% contribution from mussel biodeposits is conservative.

There was a significant difference in bacterial abundance in the top 0.5 cm of sediment in S, SM and SMSC-cores, but no significant differences were detected at 0.5–1 or 1–1.5 cm (Table 1). Bacterial abundance in surface interval was significantly greater in SMSC-cores (1.6±0.3×10^7^ cells cm^−3^) than either SM-cores (0.7±0.1×10^7^ cells cm^−3^) or S-cores (0.4±0.1×10^7^ cells cm^−3^) (Figure 1). There was no significant difference between S and SM-cores, most likely due to the high variance (C.V. = 0.33) among replicates of the SM-cores, although bacterial abundance was on average 67% greater in the top 0.5 cm of SM-cores compared to S-cores.

### Nutrient Fluxes

**Figure 3:**
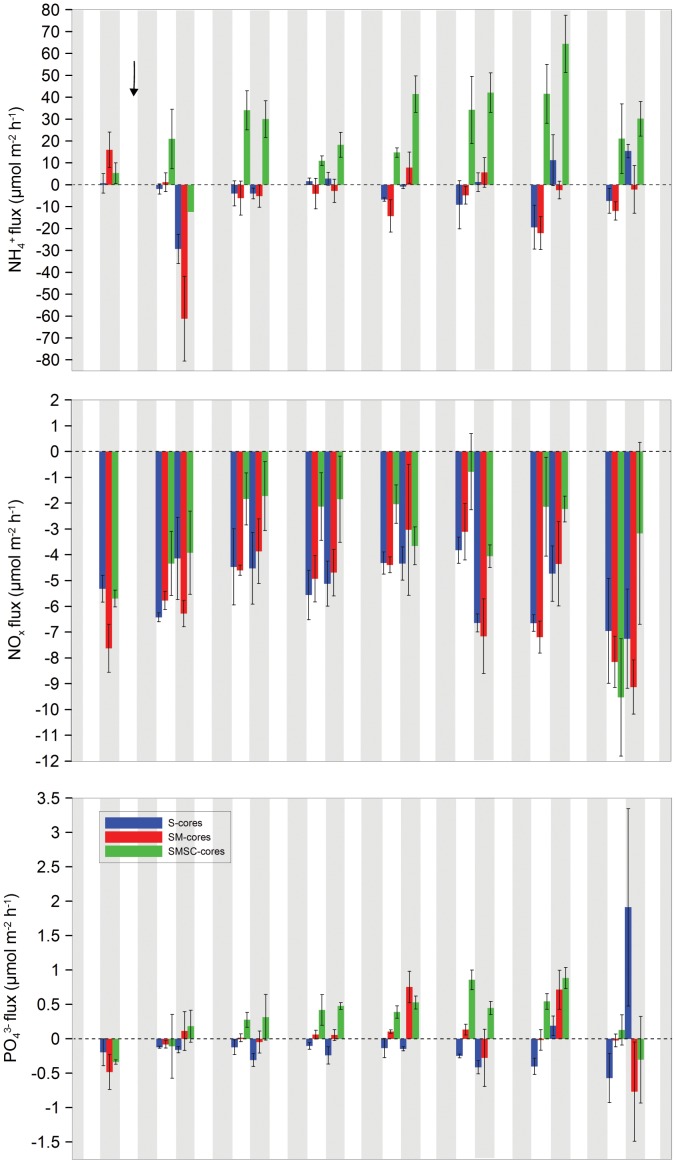
The mean flux of NH_4_^+^, NO_x_, and PO_4_^3^ ^−^ **(µmol** **m** ^−**2**^ **h** ^−**1**^ **) for each treatment on each of the experimental days.** Error bars represent the standard error about the mean. The black arrow illustrates when the first biodeposits were added. White areas signify light hours and grey areas dark hours.

There was no significant difference in sediment–seawater fluxes of NH_4_^+^, NO_x_ or PO_4_^3−^ among treatments before the experiment (Table 1). Two cores had anomalously high NH_4_^+^ flux rates before and during the experiment and had to be removed from the NH_4_^+^ analysis to meet statistical assumptions. NH_4_^+^ flux varied significantly between experimental days and across treatments, but not between light/dark measurements (Table 1). There was also a significant interaction between experimental day and light/dark and experimental day and treatment, most likely due to the elevated NH_4_^+^ influx across treatments on Day 2 of the experiment (Figure 3). NH_4_^+^ efflux was significantly greater in SMSC-cores than in S and SM-cores, which exhibited no significant difference from each other (Table 1). Rates of NH_4_^+^ flux in the S and SM-cores remained similar across the 14-day experiment, with mean fluxes of 8 to −22 µmol m^−2^ h^−1^ in SM-cores and 15 to −19 µmol m^−2^ h^−1^ in S-cores. Conversely, aside from the dark hours of Day 1, SMSC-cores demonstrated a mean efflux of NH_4_^+^ with mean efflux rates of 11–64 µmol m^−2^ h^−1^.

The flux of nitrite/nitrate (NO_x_) varied significantly between experimental days but not across treatments or between light and dark hours (Table 1). There was also a significant interaction between experimental day and light/dark, likely due to the lower influx of NO_x_ during the light hours of Day 9 and/or the significantly higher rates of NO_x_ influx on Day 14 in all treatments, particularly in SMSC-cores (Table 1, Figure 3). Although variation among treatments was not significant, NO_x_ influx tended to be lowest in SMSC-cores. There was no significant difference between S and SM-cores. When Day 14 was removed and the analysis re-run, variation between treatments remained insignificant (Repeated Measures ANOVA, *F*_2, 9_ = 3.27, *p* = 0.086).

The flux of phosphorus (PO_4_^3−^) varied significantly between light and dark, but not between experimental days or treatments (Table 1). There was a significant interaction between experimental day and treatment; and experimental day, treatment, and light/dark, most likely due to Day 14. Diurnal patterns and differences among treatments differed considerably from those on Days 2–12 (Figure 3). When Day 14 was excluded, PO_4_^3−^ flux rates varied significantly between both experimental days and treatments (Table 1). There were significant interactions between experimental day and light/dark and experimental day and treatment. PO_4_^3−^ fluxes on Day 8 were significantly different to those on Days 2 and 4. Rates of PO_4_^3−^ efflux increased in SMSC-cores as the experiment progressed (Linear Regression, R^2^ = 0.730, *p*<0.001), which probably explains the significant interactions. Further, this increase together with a spike in PO_4_^3−^ efflux in SM-cores on Day 8, likely explains the significance of experimental day. PO_4_^3−^ fluxes varied significantly between S and SMSC-cores, but not between S and SM or SM- and SMSC-cores. There was a PO_4_^3−^ influx in S-cores during all but the dark hours of Day 12, whilst there was a mean PO_4_^3−^ efflux of >0.2 µmol m^−2^ h^−1^ in SMSC-cores during all but the light and dark hours of Day 2. Although there was no significant difference between SM and SMSC-cores, the mean efflux from SM-cores only exceeded 0.2 µmol m^−2^ h^−1^ twice from Day 2–12.

### Sediment Oxygen Dynamics

Significant differences between light and dark hours for total O_2_ exchange (TOE), depth integrated O_2_ consumption/production (*R*) and O_2_ penetration depth (OPD) demonstrate the importance that microphyte photosynthesis had across all treatments in this experiment (Table 1, 2). No significant difference in *R* was found between I and S-cores during either light or dark hours (Table 1). Likewise, the OPD’s in I and S-cores were similar during both light and dark hours, ranging from 5.1–8.8 mm in S-cores and from 6.1–9.3 mm in I-cores.

**Table 2:** Comparisons of the oxygen penetration depth (OPD), total (TOE) and depth-integrated O_2_ consumption rates (*R*) for each treatment (I, S, SM and SMSC-cores) during dark hours and light hours.

	Dark hours			Light hours		
Treat.	R	TOE	OPD	R	TOE	OPD
I	−162±27	–	8.6±0.7	823±191	–	10.7±0.8
S	−202±30	−827±41	7.8±0.6	648±202	849±300	10.2±0.3
SM	−353±50	−1686±64	4.7±0.4	853±121	586±222	6.8±0.1
SMSC	−390±48	−1411±123	4.0±0.4	623±396	−229±191	6.1±0.4

All rates of O_2_ consumption/production are in µmol m^−2^ h^−1^. Note: positive values indicate production and negative values consumption.

The addition of biodeposits had a significant effect on sediment O_2_ dynamics. The OPD was significantly reduced in SM-cores in comparison to S-cores, and, during dark hours, TOE (consumption) was significantly higher in SM-cores than S-cores (Table 2). During light hours, however, no significant difference was found in TOE or *R* between S and SM-cores (Table 1). No significant difference in *R* was detected between S and SM-cores during dark hours. Nonetheless, the mean *R* in SM-cores was considerably higher than in S-cores, and there was no overlap in the data. Thus, a significant difference may have been obscured by low replication.

Neither TOE during dark hours nor *R* during light hours differed significantly between SM and SMSC-cores (Table 1). In spite of this, the mean TOE in SM-cores during light hours was considerably higher than that of SMSC-cores–SM-cores exhibiting net O_2_ production (586±222 µm m^−2^ h^−1^) and SMSC-cores net O_2_ consumption (−229±191 µm m^−2^ h^−1^) (Table 2). Furthermore, there was no overlap in the data, suggesting that low replication may have again obscured any significant difference. The mean *R* during light hours was slightly lower in SMSC-cores than in SM-cores although SMSC-cores had a considerably larger standard error. Indeed, two SMSC-cores exhibited *R* values (1202 and 1238 µmol m^−2^ h^−1^) higher than in any SM-core whilst the other two were lower than in any SM-core (499 and −449 µmol m^−2^ h^−1^). During dark hours *R* was slightly higher in SMSC-cores than in SM-cores and the OPD (during both light and dark hours) was slightly shallower in SMSC-cores than in SM-cores, however these differences were not significant.

Comparisons between *R* and TOE suggest that diffusive O_2_ exchange contributed most to TOE in SMSC-cores. During dark hours, *R* accounted for 24% of the mean TOE in S-cores, 21% in SM-cores and 28% in SMSC-cores. During light hours, *R* accounted for 76% of TOE in S-cores and 146% in SM-cores. The percentage could not be quantified for SMSC-cores because TOE was negative and *R* positive, but suffice to say that *R* indicated considerably higher production than TOE.

## Discussion

The deposit-feeding sea cucumber Australostichopus mollis suppressed benthic microalgae and facilitated bacterial activity, thus causing a shift in the balance of benthic production and decomposition processes. The increased efflux of inorganic nitrogen from the sediments in the presence of the sea cucumber promoted algal productivity; however, grazing of the microphytes by the sea cucumbers had a net negative effect on primary producer biomass. The activities of this species enhanced bacterial abundance, which facilitated mineralization processes and provides a mechanistic explanation for the observed reduction in sediment OM content. These changes in geochemistry exemplify the significant role sea cucumbers can play in ameliorating the effects of eutrophication and influencing nutrient cycling in coastal ecosystems.

### Sea Cucumbers Stimulate Bacteria and Facilitate OM Decomposition

The deposit-feeding sea cucumbers increased bacterial abundance and OM remineralization rates in coastal sediments, thus providing a mechanism for enhanced OM decomposition in the presence of bioturbators. The addition of mussel biodeposits significantly increased OM content (Figure 1) and pigment concentration (Figure 2) in surface sediment, consistent with previous studies [30], [31], [32]. However, OM content was reduced and nutrient efflux significantly elevated in sediment with *A. mollis,* suggesting increased decomposer activity. Bacterial counts in the sediment surface (Figure 1) and diurnal patterns in O_2_ exchange (Table 2) demonstrate that *A. mollis* had a positive effect on bacterial abundance. *A. mollis* respiration consumed only −284±64 µm m^−2^ h^−1^ relative to an O_2_ demand of −1,411±123 µm m^−2^ h^−1^ (Table 2) for other metabolic processes including bacterial decomposition. The stimulatory effects of benthic infauna (e.g., polychaetes) on bacterial activity are well documented [21], [33], [34], with positive interactions attributed to the removal of inhibitory metabolites and the increased aerobic sediment area associated with burrows and irrigation activity of infauna [21]. Here we demonstrate that surface deposit-feeding holothurians also stimulate bacteria through their reworking and mixing of sediment particles (bioturbation) [35].

In addition, *A. mollis* may have enhanced bacteria through deposition of feces in surface sediments. Fresh feces of holothurians are enriched in organic matter (indicated by pigment concentrations; Figure 2) and bacteria [36]. Moreover, the reduced diffusive distances between food sources within these feces, and its enhanced degradation state [37], may facilitate bacterial proliferation [32]. We propose that by reworking and reconstituting sediment, *A. mollis* may provide preferable surface areas for microbial decomposers, thus explaining their net positive effect on bacterial abundance. When coupled with evidence that bacteria may also have a positive effect on sea cucumbers–by enhancing the ‘bioavailability’ of less labile organic matter [35], it is clear that sea cucumber-bacterial interactions warrant further research.

Overall, sediment OM content was significantly reduced in the presence of *A. mollis,* despite the high OM content in fresh *A. mollis* feces and elevated bacterial abundance. While a higher proportion of sediment OM content would have comprised bacteria in SMSC-cores, bacteria have a maximum growth efficiency of around 50% [38]. Thus, <50% of organic matter assimilated by bacteria is retained as sediment OM in the form of new bacterial biomass, while the remainder is respired in inorganic form. Accordingly, the presence of the microbial decomposers and the reduced assimilation of remineralized nutrients by producers (see below) resulted in a net overall decrease in sediment OM content in the presence of *A. mollis*. While previous studies have argued the potential of sea cucumbers to counter eutrophication directly through their own energy requirements [7], [8], our study demonstrates that sea cucumbers can also ameliorate the effects of eutrophication indirectly through facilitation of bacterial decomposers.

The possibility that *A. mollis* created favorable conditions for bacteria by inducing changes in the benthic meiofauna should also be considered as bioturbation can negatively impact some meiofauna [39]. The current study does not include meiofauna as this was not the focus of our study, and thus we cannot assess the role of meiofauna on bacteria. Regardless of the specific mechanism, our findings demonstrate that bacteria were stimulated in the presence of omnivorous deposit-feeding sea cucumbers and thus support the theory that bacterial facilitation may be important for the persistence of omnivory in some benthic communities [17].

The addition of biodeposits [32] and bioturbation [1] can affect sediment geochemistry and nutrient exchange between the sediment and overlying seawater. Bioturbators can increase the efficiency of diffusive solute transport [40] due to interphase mixing [41], which may have contributed to the elevated release of nutrients in SMSC-cores. However, as no significant difference was detected in porosity between SM and SMSC-cores, there is no immediate support for this idea. Rather, bacterial counts (Figure 1) and oxygen data (Table 2) demonstrate that bacteria proliferated in the presence of sea cucumbers and this promoted OM decomposition and affected benthic–pelagic exchange of nutrients. The influx of NO_x_ tended to be lower when *A. mollis* was present (Figure 3), while there was increased availability of NH_4_^+^ that would aid nitrification (bacteria-mediated oxidation of NH_4_^+^ to NO_x_) and decrease the demand of denitrifiers for external sources of NO_x_. With readily available NH_4_^+^, O_2_ may be the main factor limiting nitrification, perhaps explaining the reduced influx of NO_x_. The presence of *A. mollis* tended to increase the efflux of PO_4_^3−^, which, until Day 14, demonstrated a significant positive correlation with time. In oxic conditions PO_4_^3−^ is readily absorbed by iron oxides [42]. Thus, the *A. mollis*-induced producer–decomposer shift would be expected to reduce O_2_ availability and hence promote the efflux of PO_4_^3−^. Clearly through direct producer–decomposer interactions, *A. mollis* also indirectly influenced a number of other sediment biogeochemical processes including benthic-pelagic nutrient cycling. The range and complexity of bioturbator-sediment biogeochemical interactions documented in our study is consistent with that of previous authors [1], [2] and reiterates that diminished bioturbator abundance is likely to have a considerable impact on sediment geochemistry and ecology in coastal ecosystems [6], [2].

### Sea Cucumbers Influence Producer Biomass Directly and Indirectly

The presence of deposit-feeding sea cucumbers directly influenced producer biomass through bioturbation and grazing, and indirectly influenced nutrient fluxes that affected microphytes (Fig. S1). The presence of *A. mollis* had a positive effect on NH_4_^+^ efflux from the organically enriched sediment during both light and dark hours (Figure 3). As NH_4_^+^ is an animal excretory product [23], the direct metabolic activity of *A. mollis* contributed to the observed NH_4_^+^ efflux. However, these bioturbators also indirectly promoted efflux of nitrogen from the sediments by facilitation of bacterial decomposers that enhanced mineralization rates (see above). Without the sea cucumbers (SM-cores), benthic producers and decomposers maintained a tight balance, with MPB biomass increasing in accordance with enhanced decomposer biomass so that rates of NH_4_^+^ efflux remained negligible (Figure 3). However, when sea cucumbers were added to the cores, grazing and bioturbation suppressed MPB, which further promoted NH_4_^+^ efflux from the sediments into the overlying seawater.

Nutrient subsidies from biodeposits enhanced benthic algal biomass (Figure 2; Fig. S2), but grazing by sea cucumbers resulted in a net reduction in MPB biomass at high densities of *A. mollis*. Indeed, NH_4_^+^ flux approximated zero in cores with biodeposit additions (SM-cores), suggesting assimilation of inorganic nitrogen by the microphytes on the sediment surface. Moreover, in spite of elevated O_2_ consumption in SM-cores (Table 2), there was no significant difference in the total net O_2_ production in S and SM-cores–a clear indication of greater MPB biomass in sediment with biodeposits.

Grazing by the deposit feeding sea cucumbers (indicated by high pigment concentrations in *A. mollis* feces; Figure 2) significantly reduced surface sediment algal biomass (Figure 1), and outweighed any positive sea cucumber–microphyte interactions (e.g., increased nutrient availability). By contrast, some studies have documented a net positive effect of deposit-feeding bioturbators (e.g., *Echinocardium* sp.) on microphytes [2], [43]. This may relate to species-specific differences in resource use [44], which would caution against classifications such as ‘deposit-feeding bioturbator’ when predicting functional roles of benthic organisms.

Bioturbator density may also explain the disparity between our findings and that of some previous studies since bioturbator density can have a non-linear effect on microbial interactions [45]. This experiment used high sea cucumber densities (∼1500 g m^−2^) as A. mollis has good survivorship at this density in organically-enriched sediments [7] and since we wanted to test their potential for intensive aquaculture applications. There was a net increase in the biomass of sea cucumbers during our experiment, indicating that the density used and the experimental conditions did not adversely affect the physical condition of A. mollis. Other studies, by contrast, have used lower densities of bioturbators. Alternatively, our findings may demonstrate the importance of productivity gradients in determining the strength of the effect of sea cucumber–microphyte interactions. Plant community interactions can become less positive, or even negative as the environments fertility increases [26], [46]. A similar ‘dynamic’ relationship may exist between bioturbators and MPB, whereby the increased nutrient availability associated with bioturbation becomes less important for MPB in nutrient rich sediments, although this mechanism would require further investigation.

The ability of microphytes to control regeneration of nutrients following significant deposition of algal detritus has not been demonstrated to date. Our findings demonstrate that MPB were stimulated by the addition of mussel biodeposits, and this increased algal biomass in turn, influenced the benthic–pelagic exchange of nutrients (see above). High MPB activity can starve nitrifiers of NH_4_^+^, driving denitrifiers demand for external sources (influx) of NO_x_ [47]. Any PO_4_^3−^ not assimilated directly by MPB likely bonded with iron and manganese oxides in the O_2_ saturated pore spaces that result from MPB photosynthesis. Given the importance of phytodetritus for coastal food webs and biogeochemical cycles [48], the relationship between pelagic productivity, deposition, and benthic productivity is an important area for future research.

### Conclusions

By facilitating bacterial abundance and suppressing microphytobenthos, deposit-feeding sea cucumbers shift the microbial balance in organically enriched marine sediments and redistribute dissolved nutrients from the sediments into the pelagic environment. The associated ecosystem-level effects–significant changes in nutrient cycling and sediment OM content, demonstrate that sea cucumbers play an important functional role in the ecology of coastal ecosystems and may be used to counter eutrophication effects of finfish or bivalve farms.

## Materials and Methods

### Study Design

Sixteen intact sediment cores (ID = 8.4 cm, acrylic tube length = 30 cm) were collected by SCUBA at 10 m (T = 10.5°C, S = 32) in Four Fathom Bay (173°52.5'E, 41°09.1'S), Marlborough Sounds, New Zealand, at a site unaffected by aquaculture farms and where *A. mollis* is commonly found. The cores were transported on ice in the dark to the laboratory where they were connected to a stirred flow-through system in a controlled temperature (CT) room set at 10°C and with a 12∶12 light/dark cycle. The photosynthetic active radiation (PAR) incident to the sediment core surface was 12 µmol quanta s^−1^ m^−2^, simulating in situ conditions [30]. Cores were left to settle for five days and the experiment conducted over the following 14 days. Seawater was maintained at 10±1°C and supplied to each core at a rate of 50±5 ml min^−1^. The overlying seawater in each core was stirred with a motor-driven magnetic rod to ensure a homogeneous, oxygenated water column that did not resuspend the sediment.

Fresh mussel feces and pseudo-feces (feces hereafter) were collected from farmed mussels (*Perna canaliculus*). The flow-through system was temporarily turned off and 5.1±1 g wet weight (w/w) homogenized feces were added to the overlying water column of eight cores on day 1 and every second day for 14 days of the experiment. Total feces additions to each core equated to 425 g w/w m^−2^ d^−1^, which approximates the median deposition rate beneath bivalve farms [29].

Nutrient fluxes were measured in all 16 cores before the experiment. Initial O_2_ concentration microprofiles and sediment characteristics were determined on four cores (I-cores). Four of the remaining 12 cores were randomly selected as control cores with sediment only (S-cores), whilst the other eight cores were treated with mussel feces as described above. Four of these cores only received mussel feces (SM-cores) and four also received cultured juvenile (3–6 cm) sea cucumbers, *Australostichopus mollis* (SMSC-cores). We used similar densities of sea cucumbers trialed under mussel farms [7]: between five and six *A. mollis* individuals, which equated to a biomass of 9.2–9.5 g added to each SMSC-core. Biomass of sea cucumbers was determined at the start and end of the experiment after starvation for 48 hours.

Nutrient fluxes were measured on alternate days to feces additions during the experiment. Total O_2_ exchange (TOE) was measured in three randomly selected cores, one from each treatment. At the end of the experiment, the S, SM, and SMSC-cores were profiled for sediment O_2_ concentration, drained, and sub-cored to determine sediment characteristics.

### Sediment O_2_ Profiling

Sediment pore water O_2_ microprofiles were measured at 100 µm increments from above the diffusive boundary layer to a maximum depth of 8 mm using a micromanipulator and a Clark-type microelectrode (Unisense) [49]. Three O_2_ microprofiles were measured in each of the 16 cores during both light and dark hours. Light measurements began four hours after illumination and dark measurements began when the lights had been off for four hours. For consistency, replicate microprofiles were done in a triangular convention, with each profile approximately halfway between the center of the core and the side. In SMSC-cores, *A. mollis* feces were avoided. Any profiles that passed through burrows (steep increase in O_2_ concentration in anoxic sediment layer) were excluded from the analyses. The mean O_2_ penetration depth (OPD) and depth-integrated O_2_ consumption/production (*R*) was calculated for each core using PROFIX software. *R* was computed using the porosity and the curvature of the line of best fit for each cores mean O_2_ profile [50].

### Sediment Characteristics

Two sub-cores (ID = 2.6 cm) were taken from each core, sectioned at 0–0.5, 0.5–1, 1–2, 2–4, and 4–6 cm intervals, to measure porosity, total organic matter (TOM) and chloropigment (chlorophyll a and pheopigment, its degradation products). A third sub-core was taken from S, SM, and SMSC-cores and sectioned at 0–0.5, 0.5–1 and 1–1.5 cm to quantify bacterial abundance. A sample of *A. mollis* feces was taken from each of the SMSC-cores and analyzed for chloropigment concentration. Samples of homogenized mussel biodeposits were taken on the first and last days of the experiment and also analyzed for chloropigment. Chloropigment, chlorophyll a and pheopigment concentrations were quantified spectrophotometrically and TOM was quantified using loss-on-ignition (500°C for 5 hours). Porosity was calculated as described by [40].

### Bacterial Abundance

Bacteria were isolated from the sediment following a modified method of [51]. Three milliliters of sterilized seawater were added to each sediment sample, sonicated (60 s), shaken vigorously and left to settle (30 s), and this procedure repeated three times. 3 ml of supernatant was filtered through a 5 µm filter and distributed evenly into three cryovials that were frozen in liquid nitrogen and stored in a −80°C freezer. These three sub-samples were counted and the mean bacterial counts (n = 3) used for statistical analyses.

Bacterial counts were determined by flow cytometry using a FACSCalibur instrument (Becton Dickinson). The sheath fluid was 0.2 µm filtered, de-ionized water and the analyzed volume was calculated using Trucount™ (Becton Dickinson) beads as a tracer. Bacteria samples were stained using SYBRII stain at a concentration of 10^−4^ of stock solution and then incubated in the dark for 10–15 minutes before being analyzed following the methods described by [52].

### Nutrient and O_2_ Solute Exchange Rates

Nutrient flux and O_2_ measurements were conducted during light and dark hours, except the pre-experiment nutrient measurements that were conducted only in the dark. As for O_2_ microprofiles, light measurements began four hours after illumination, and dark measurements began when the lights had been off for four hours. Solute exchange rates were quantified using core incubations (3.5–6 h) and taking water samples at the beginning and end of the incubation period to measure nutrient (25 ml) and dissolved O_2_ (60 ml) concentrations. Nutrient samples were immediately filtered (Whatman GF/C) and frozen at −20°C before being analyzed within one month for NO_x_ (as NO_3_^−^+NO_2_^−^), NH_4_^+^ and PO_4_^3−^ using a QuikChem 8000 Automated Ion Analyzer (Lachat Instruments Inc., Milwaukee, WI, USA). The Winkler method was used to determine O_2_ concentrations.

To isolate the effect of *A. mollis* on the sediment O_2_ consumption, we measured their respiration after the 14-day experiment. After starvation and weighing, the sea cucumbers were fed mussel feces in the same quantities used during the experiment. Each group of sea cucumbers was transferred into a core containing sediment void of OM (ashed at 500°C for 5 h). Using the same procedure as for O_2_ flux measurements, the rate at which each group of sea cucumbers consumed O_2_ was quantified during both light and dark hours. The values were used to correct the TOE in SMSC-cores. Oxygen production values are presented as positive fluxes and consumption as negative fluxes.

### Statistical Analysis

One-way ANOVAs (α = 0.05) were used to compare sediment characteristics across the four treatments at each depth interval. One-way ANOVA analyses were used to compare chloropigment in *A. mollis* feces, mussel feces, S, SM, and SMSC-cores and to test for differences in nutrient concentrations among I, S, SM and SMSC-cores before the experiment. For nutrient data collected after mussel feces additions, Repeated Measures ANOVA tests were used with experimental day and day/night (diurnal) as the repeated measures factors, and core type as the third factor. Repeated Measures ANOVAs were also used to test for significant differences in OPD, *R*, and TOE with day/night as the repeated measures factor and core type as the other factor. When significant interactions were detected, one-way ANOVAs were employed. Assumptions of normality and equality of variances were tested using Levene’s test of equality and residual plots. Any data points identified as outliers by residual plots were further investigated, and when appropriate, eliminated from the analysis. Some sediment characteristics data were log_10_ transformed to meet the assumptions of normality.

## Supporting Information

Figure S1 **Schematic depicting the direct (solid lines) and indirect (dashed lines) effects of the sea cucumber,** ***Australosticopus mollis*** **, on remineralization and nitrogen efflux from the sediments, microphytobenthos biomass and bacterial abundance.** The direction of the interaction, positive (+) or negative (−) is indicated and stronger interactions are illustrated in bold arrows.
(TIF)

Figure S2 **The sediment surface of a core (A) with no biodeposits only (S-core), (B) with biodeposits (SM-core) and (C) with biodeposits and sea cucumbers (SMSC-cores).** Microphytobenthos (MPB) (shown by a copper surface film) were stimulated following biodeposit additions (B) but were visually disrupted in the presence of epibenthic sea cucumbers.
(EPS)
