# Supplementary figures and images for: Deposit-Feeding Sea Cucumbers Enhance Mineralization and Nutrient Cycling in Organically-Enriched Coastal Sediments
Source: PLoS One. 2012 Nov 27;7(11):e50031. doi: 10.1371/journal.pone.0050031 (PMC3507890; doi:10.1371/journal.pone.0050031)

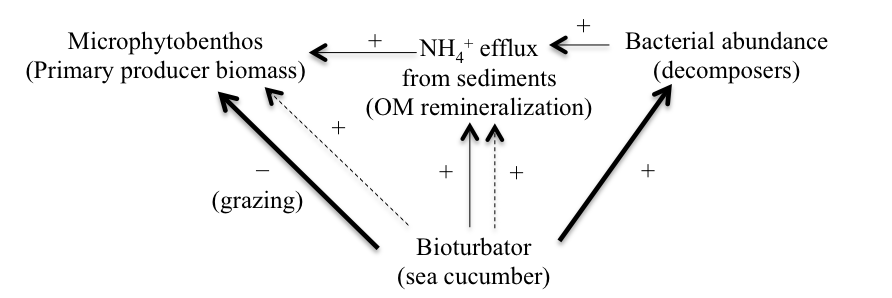

Supplement: Figure S1 — Schematic depicting the direct (solid lines) and indirect (dashed lines) effects of the sea cucumber, Australosticopus mollis , on remineralization and nitrogen efflux from the sediments, microphytobenthos biomass and bacterial abundance. The direction of the interaction, positive (+) or negative (−) is indicated and stronger interactions are illustrated in bold arrows. (TIF) [file pone.0050031.s001.tif]
